# New Perspectives on the Ecology and Evolution of Siboglinid Tubeworms

**DOI:** 10.1371/journal.pone.0016309

**Published:** 2011-02-14

**Authors:** Ana Hilário, María Capa, Thomas G. Dahlgren, Kenneth M. Halanych, Crispin T. S. Little, Daniel J. Thornhill, Caroline Verna, Adrian G. Glover

**Affiliations:** 1 Centro de Estudos do Ambiente e do Mar and Departamento de Biologia, University of Aveiro, Aveiro, Portugal; 2 Australian Museum, Sydney, Australia; 3 Zoological Department, University of Gothenburg, Goteborg, Sweden; 4 Department of Biological Sciences, Auburn University, Auburn, Alabama, United States of America; 5 School of Earth and Environment, University of Leeds, Leeds, United Kingdom; 6 Department of Biology, Bowdoin College, Brunswick, Maine, United States of America; 7 Symbiosis Group, Max Planck Institute for Marine Microbiology, Bremen, Germany; 8 Zoology Department, The Natural History Museum, London, United Kingdom; Ecole Normale Supérieure de Lyon, France

Siboglinids are tube-dwelling annelids that are important members of deep-sea chemosynthetic communities, which include hydrothermal vents, cold seeps, whale falls and reduced sediments. As adults, they lack a functional digestive system and rely on microbial endosymbionts for their energetic needs. Recent years have seen a revolution in our understanding of these fascinating worms. Molecular systematic methods now place these animals, formerly known as the phyla Pogonophora and Vestimentifera, within the polychaete clade Siboglinidae. Furthermore, an entirely new radiation of siboglinids, *Osedax*, has recently been discovered living on whale bones. The unique and intricate evolutionary association of siboglinids with both geology, in the formation of spreading centres and seeps, and biology with the evolution of large whales, offers opportunities for studies of vicariant evolution and calibration of molecular clocks. Moreover, new advances in our knowledge of siboglinid anatomy coupled with the molecular characterization of microbial symbiont communities are revolutionizing our knowledge of host-symbiont relationships in the Metazoa. Despite these advances, considerable debate persists concerning the evolutionary history of siboglinids. Here we review the morphological, molecular, ecological and fossil data in order to address when and how siboglinids evolved. We discuss the role of ecological conditions in the evolution of siboglinids and present possible scenarios of the evolutionary origin of the symbiotic relationships between siboglinids and their endosymbiotic bacteria.

## Introduction

**Figure 1:**
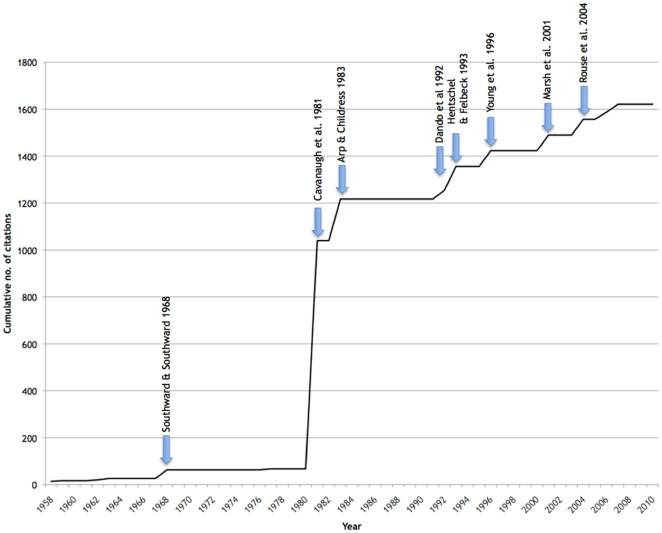
Citation counts for papers published in the journals *Nature* or *Science*. Cumulative citation count for papers published in over the years 1958 to 2007 that deal exclusively with species in the annelid clade Siboglinidae (papers covering general vent/seep biology or symbiosis in general are not included). Significant discoveries are highlighted by arrows and major increases in total citations. These include discoveries in feeding [10], the discovery of bacterial symbiosis [13], sulfide binding [18], tubeworms at shipwrecks [20], respiration [22], embryology [23], larval dispersal [24] and the new clade of siboglinids (*Osedax*) that consume whale bones [25].

**Figure 2:**
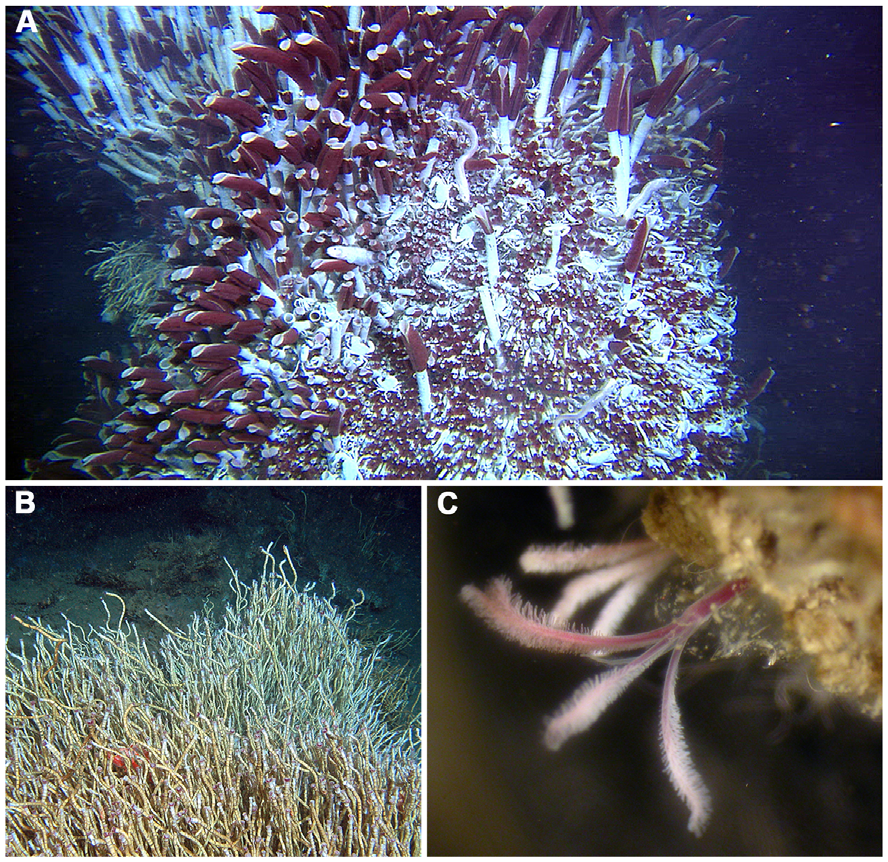
Examples of siboglinid species and their habitat requirements. A) *Riftia pachyptila* giant tubeworms growing on a hydrothermal vent in the north-east Pacific (Image courtesy of Richard Lutz), B) *Lamellibrachia luymesi* at a cold seep in the Gulf of Mexico (Image courtesy of DT, KH, Kevin Fielman and Scott Santos) and C) *Osedax mucofloris* living on a whale-bone found off the coast of Sweden.

Deep-sea worms in the polychaete family Siboglinidae are not yet thought to be of any commercial or medical value to humans. Nevertheless, in 50 years of research, 27 publications have appeared in the top-cited science journals *Nature* and *Science* that deal exclusively with species in this group and these papers have been cited a total of 1621 times as of the time of writing [1]–[27] (Figure 1). The highest-cited paper (for which metrics exist) on any siboglinid [13] has received 389 citations, 147 more than the next highest-cited paper in that same issue of *Science*, on the role of insulin in determining diabetes [28]. Not surprising, 13 of these 27 publications in *Nature* or *Science* focus exclusively on a single species of siboglinid worm, *Riftia pachyptila* Jones, 1980 (Figure 2a). This giant worm, discovered on hydrothermal vents at the Galapagos Rift in 1977 became the poster-child of deep-sea discovery, the ‘lost world’ of unknown animal lineages that scientists on the Challenger deep-sea expedition 100 years previously had so wanted, but failed, to find. Arguably, this single species of worm launched the careers of a generation of deep-sea biologists.

Taxonomy and systematics have played a crucial, but unsung, role in the elevation of these discoveries to the international media. Early deep-sea biologists, the ‘Challenger generation’, were desperate to discover living fossils in the deep – trilobites crawling through abyssal muds, the lost world of the Mesozoic in the dark depths of the ocean. Thus some may have been disappointed to discover that although life was abundant and diverse in the deep sea, the majority of species were in the same families, and often congeneric with shallow-water forms. Hence the discovery of a new group of deep-sea creatures [29] and the creation of a new phylum, Pogonophora [30] grabbed media headlines in the 1950s [31], as did the discovery of a new family of Pogonophora, the Riftiidae, on hydrothermal vents in the 1970s [15]. Under much controversy [32], *Riftia pachyptila* was elevated to phylum ‘status’ [33] under the name Vestimentifera. However, its status as phylum was short-lived as new methods in cladistic analyses and the arrival of molecular phylogenetics changed our understanding of evolution in the Metazoa.

A series of papers through the last twenty years has supported the placement of tubeworms as a single family (Siboglinidae) within the annelid radiation, as originally postulated by Uschakov in 1933 [34]–[40], bringing the tale of Pogonophora and Vestimentifera full circle. However, the story of Siboglinidae has, in the last five years, received a new twist: the discovery of an entirely new species-rich clade of highly derived siboglinids, known as *Osedax*, that appear to live exclusively on mammal (typically whale) bones [25], [41]–[42].

**Figure 3:**
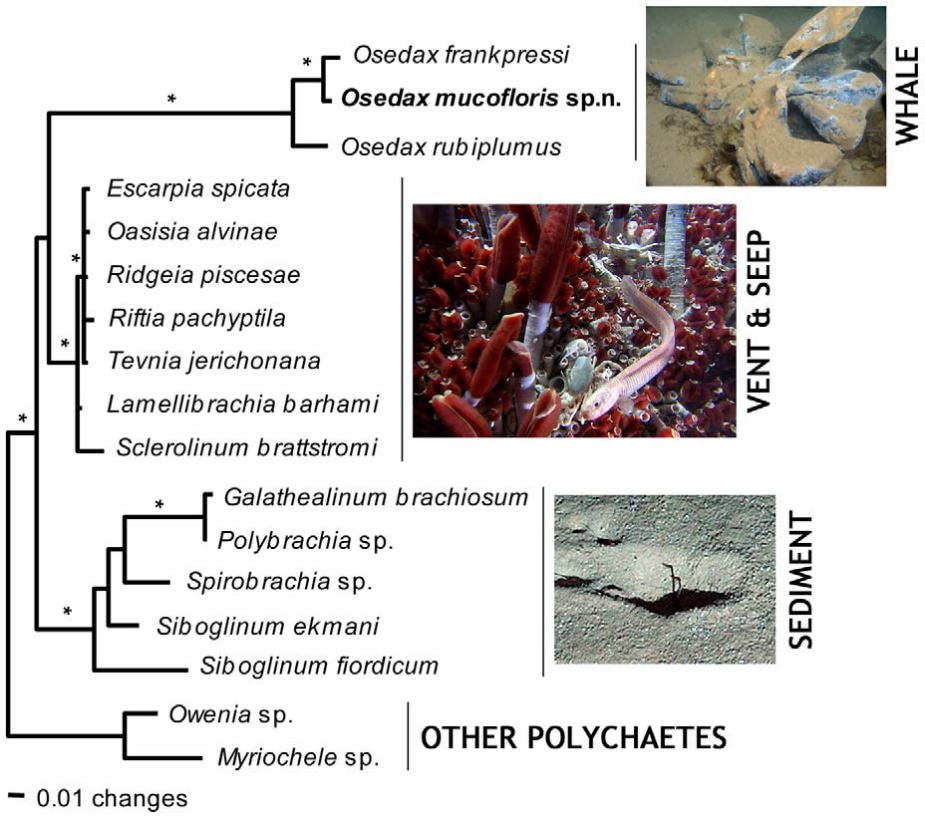
Phylogenetic relationships amongst Siboglinidae. A Bayesian analysis of 18S ribosomal RNA sequences reveals four major clades of siboglinids, from top, *Osedax* which are specialist on whale carcasses, the vestimentiferans, which are specialist on vents and seeps, *Sclerolinum* (here presented only by a single sequenced specimen), specialist on organic-rich remains and the frenulates which specialise on organic-rich sediments. Modified from [41]. Images courtesy of Tomas Lundälv (whale-fall), Richard Lutz (vent site) and NOCS/JC10 (frenulate in sediment).

**Figure 4:**
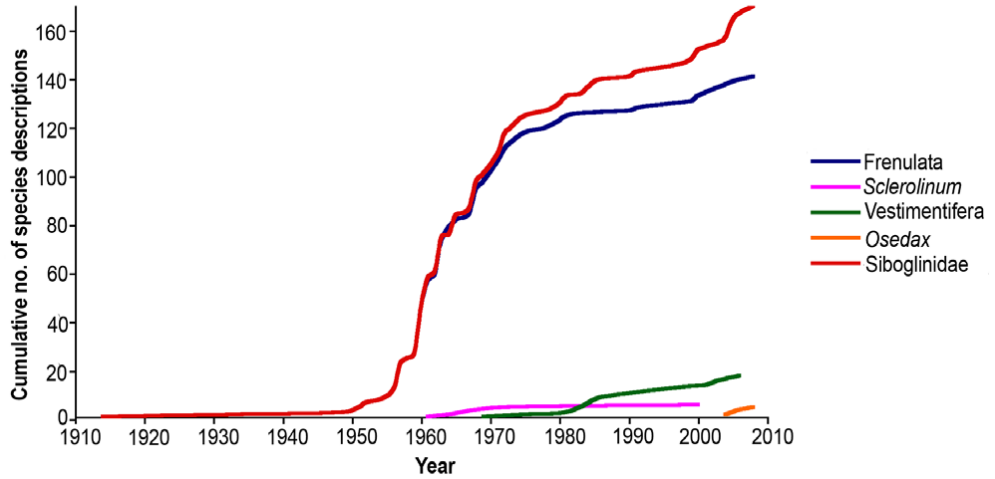
Cumulative number of species descriptions since the discovery of the first siboglind. With the exception of *Sclerolinum*, the curve does not asymptote showing that new species have been (up to this day) continuously disclosed.

Currently most researchers recognize four main lineages within Siboglinidae: Frenulata, Vestimentifera, *Sclerolinum* and *Osedax* (Figure 3). *Sclerolinum* was originally regarded as a frenulate and later placed in its own taxon, Monilifera, equal in rank to Frenulata and Vestimentifera [43]. Recent molecular and morphological studies however, show that *Sclerolinum* is the sister clade to vestimentiferans [40], [44]. Among the four siboglinidae lineages, frenulates are by far the most diverse with 141 nominal species. By contrast, vestimentiferans have 18 species, *Sclerolinum* six, and *Osedax* five (at the time of writing several new species for all groups were in the process of being described and thus the numbers are major underestimates) (Figure 4). Although biological generalizations are often problematic, each siboglinid clade is, in general, found in a certain type of habitat. Frenulates are typically found in muddy (often deep) environments; vestimentiferans typically occur in hydrothermal vent and hydrocarbon seep areas; *Sclerolinum* is known to live on organic decaying organic matter (e.g., wood and rope) but also occurs free-living in mud; whereas *Osedax* is found exclusively on vertebrate bones.

With the exception of *Osedax*, the external anatomical characters are relatively constant among all siboglinids. These worms have a chitinous close-fitting tube of their own secretion that provides both protection and support (reviewed in [45]). The body can be divided into four main regions: an anterior region, a diaphragm, a trunk region and a segmented opisthosoma. In Vestimentifera, the anterior region is called the obturaculum, it functions as an operculum that closes the tube when the animal withdraws, and supports the large branchial plume. In frenulates and *Sclerolinum* the equivalent region includes a cephalic lobe and dorsal tentacles, two in *Sclerolinum* and from one to over 200 in frenulates. The second body region is responsible for the names Vestimentifera and Frenulata. In vestimentiferans it is called the vestimental region and is characterized by two dorsolateral folds with a ciliated field on the ventral side [46]. In frenulates and *Sclerolinum*, this region is called the forepart [47] and is characterized by the presence of a cuticular structure called the frenulum and the presence of a ventral ciliated band, respectively. Adjacent to the vestimentum/forepart is the elongated trunk region in which the gonads and the trophosome, the organ that holds the symbiotic bacteria, are enclosed. In all three groups the opisthosoma is divided by septa into coelomate segments, with regularly arranged chaeta. Most of the features shared with annelids are concentrated in the opisthosoma, including muscular septa, segmentally arranged chitinous chaetae, ganglia and blood vessels (reviewed in [45]).

In contrast to other siboglinids, bone-eating *Osedax* species show a marked sexual dimorphism with dwarf paedomorphic males resembling other siboglinid larvae [25], [48], [49]. Females have a transparent mucous tube that encloses the trunk. The posterior portion of the trunk reaches into the bone and forms a complex system of “roots” that contain an ovisac covered with tissue containing endosymbiotic bacteria. Although the microscopic males are provided with chaetae on the posterior portion of the body, the females have no opisthosome, which makes the morphological affinity with annelids more difficult to recognize.

Whilst there are many unanswered questions regarding the ecology and evolution of these strange deep-sea worms, three important facts are now accepted: (1) adult siboglinids lack a gut, mouth, anus and conventional feeding ability, (2) siboglinids studied thus far possess bacterial symbionts and (3) siboglinids form a well-supported monophyletic clade.

Given the conspicuous absence of a digestive system, many functional studies of siboglinids have concentrated on the question of nutrition. Early hypotheses centred on the possibility of dissolved organic matter (DOM) uptake across the body wall [50]. The twin papers of Cavanaugh et al. [13] and Felbeck [14] revolutionized this viewpoint by showing that larger siboglinids utilized symbiosis with chemoautotrophic bacteria. Although all siboglinids are assumed to house endosymbiotic bacteria for nutrition, symbionts have only been confirmed in a small minority of the 170 described siboglinid species. Furthermore, the discovery of unexpectedly different metabolic types of symbionts, with putatively heterotrophic metabolism opposed to chemoautotrophy, in the *Osedax* clade [51] and potential symbiont diversity in other gutless worms [52] has illustrated that much knowledge of the diversity and function of these relationships awaits discovery. Most of the work on endosymbiont evolution has focused on vestimentiferans [13], [26], [53] and considerable microbiological work has already been undertaken on *Osedax* [51], [54], [55]. In contrast, endosymbionts of frenulates and *Sclerolinum* have only recently been explored [56]–[59].

The evolutionary history of siboglinids has no doubt been a complex interaction of host and microbe evolutionary trajectories. Based on molecular genetic and morphological evidence [25], [60], we may infer that over evolutionary time conventional heterotrophic polychaetes made the evolutionary leap to specialize as obligate endosymbiotic siboglinid species at chemosynthetic ecosystems. The aim of this paper is to address when and how this happened reviewing the available morphological, molecular, environmental and fossil data.

## Results

### When did siboglinids evolve?

#### Clues from phylogenetic studies

The complex taxonomic story of the siboglinids has been recently well reviewed [40], [61]–[63] and is, as Rouse [40] stated “one of the more fascinating tales in animal systematics.” In the days prior to robust cladistic analysis or molecular evidence, a long scientific debate was held as to the possible origins of these enigmatic worms. Some of the early work was suggestive of a deuterostome origin (e.g., [30], [64]) whilst others supported an annelid relationship (e.g., [34], [65]–[67]. Initially, the debate centred on whether the position of the brain and nerve cord was dorsal, which is the classical deuterostome arrangement. The problem was the lack of a reference point (a gut) for determination of the dorsal or ventral position. The discovery of the opisthosome region at the posterior end of the worm, with its clear annelid-like segmentation and serially-arranged chaetae [67], [68] should have been sufficient evidence to place the Pogonophora phylum, as it was then known, within the annelid radiation. However, supporters of the phylum designation maintained their stance for several more decades (e.g., [43], [69]).

The incredible discoveries of the late 1970s of giant worms at hydrothermal vents pushed tubeworms, Pogonophora and the new group of Vestimentifera back onto journal covers and the popular press (Figure 1 and references therein). These discoveries also re-ignited the debate as to the origins of the Pogonophora, and in particular the relationships between the Pogonophora, Vestimentifera and annelids. For a time, the vestimentiferans were elevated to phylum status [33], although later studies found close links in the larval development of both Pogonophora and Vestimentifera [32]. To some, these discussions might have appeared as obscure taxonomic arguments of little relevance to modern day issues in biology. But they are relevant to our first major question – when did siboglinids evolve? Are the siboglinids an ancient lineage that branched from the rest of the Metazoa not long after the evolution of the major animal groups? Or are they a more recently-evolved branch of the tree of life, derived from more conventional filter-feeding polychaetes with which they share several morphological similarities?

Modern systematics can provide preliminary answers to this difficult question. The first robust cladistic analysis of morphological characters in polychaete families [38] showed strong support for the placement of the pogonophorans and vestimentiferans as a clade within the polychaete group Sabellida. At a similar time, several early molecular studies also showed support for a polychaete-origin for siboglinids [37], [70]–[72]. A taxonomic revision was undertaken [40] and together with molecular studies [39], [44], [73]–[75] the name Siboglinidae is now firmly established as representative of the worms formally known as Vestimentifera and Pogonophora.

Whilst Siboglinidae as a clade of annelid worms is now well accepted, this improvement in the taxonomic situation has done little to help answer our primary question – when did siboglinids evolve? Annelida is an ancient branch of the Metazoa that has probable Lower Cambrian origins at least [76]. However, these early, putative stem-group annelids resemble the errant polychaetes Phyllodocida, characterised by their clear segmentation and well-developed parapodia and chaetae. Although support for placement within current classifications is weak [77], current evidence suggests that Siboglinidae are likely affiliated with the Oweniidae within a clade of ‘sabellimorph’ species that include the Serpulidae and Sabellidae [39], [73]. These polychaetes all share a similar sessile, tube-dwelling lifestyle and exhibit less pronounced segmentation and reduced chaetal structures. In general the fossil record of these animals is poor, with the main exception being the calcareous tube-forming Serpulidae, which have a slightly better fossil record dating back to the Late Triassic [78]. However, the presence of sabellimorph, tube-dwelling polychaetes in the fossil record does little to help narrow the window of geological history during which Siboglinidae may have evolved.

Molecular genetics can help. In theory, genetic differences between closely related taxa allow the establishment of a divergence time based on a known rate of accumulation of neutral genetic differences (the molecular clock). Intriguingly, the few studies of molecular clocks in annelids come from studies of Siboglinidae. The first attempt to age the Siboglinidae based on genetic data suggested a relatively recent Mesozoic or Cenozoic origin [70]. Molecular clocks for Siboglinidae can, in some instances, be calibrated as hydrothermal vent species are intrinsically linked with geology as mid-ocean ridges form and separate. A calibration of the molecular clock for siboglinid and ampharetid polychaetes, made using the genetic divergence between closely related species living on two different mid-ocean ridge systems, also suggested a recent origin of approximately 60 mya [79]. Apart from one other older estimate (126 mya [80], [81]), work in this area has since stalled and more recent studies have focused mainly on direct evidence from fossils.

#### Clues from the fossil record

**Figure 5:**
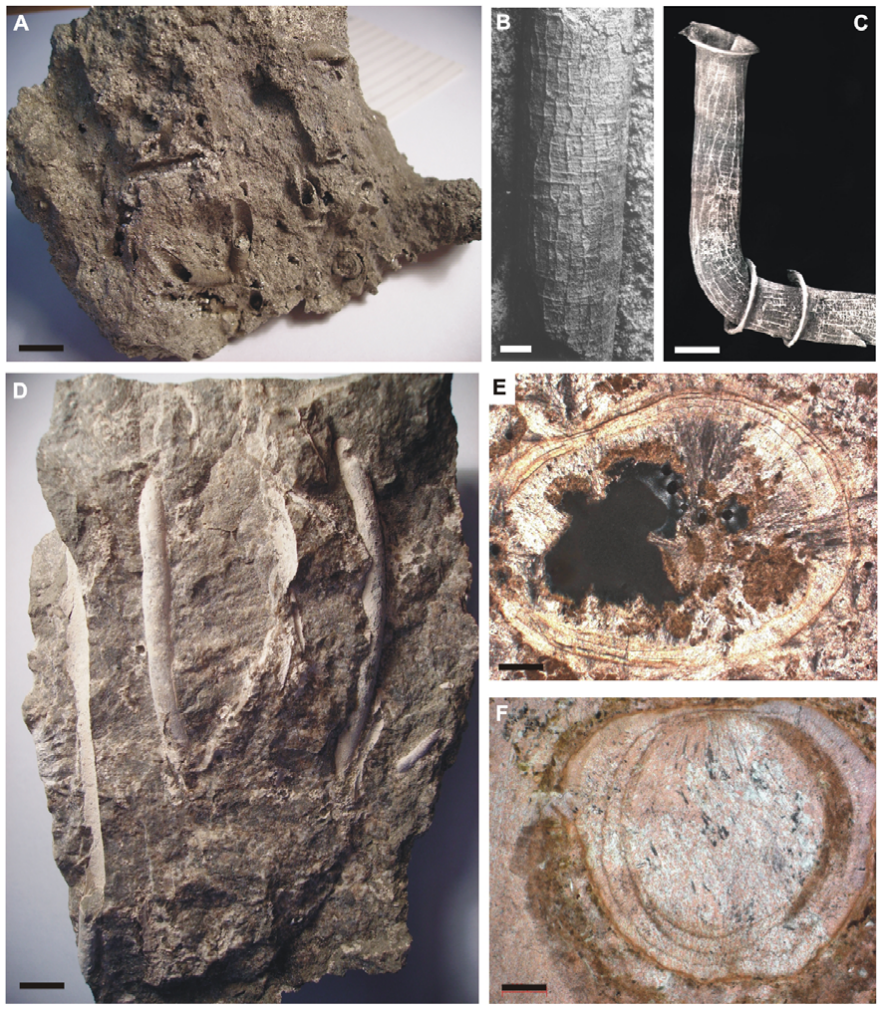
Tube fossils possibly attributable to vestimentiferans. Tube fossils from ancient seep and vent deposits possibly attributable to vestimentiferans and modern vestimentiferan tubes for comparison. A) Cluster of pyrite replaced tubes in matrix of pyrite, Kambia vent deposit, Cyprus, Early Cretaceous (91 Ma). B) Pyrite replaced tube in pyrite matrix, Figueroa vent deposit, California, USA, Early Jurassic (∼184 Ma), note fine concentric growth lines and wavy, periodically bifurcating longitudinal ridges. C) Tube of holotype (NHM1996:1048) of vestimentiferan *Arcovestia ivanovi*, note external ornament of fine concentric growth lines and wavy, periodically bifurcating longitudinal ridges. D) Carbonate tubes in matrix of carbonate minerals, Canyon River seep deposit, Washington, USA, Oligocene (∼30 Ma), specimen courtesy of James Goedert. E) Carbonate replaced tube of vestimentiferan (probably *Escarpia southwardae*) in transverse section from modern seep in the Kouilou pockmark field on the Congo deep-sea fan, 3100m water depth. The original organic tube has been ‘delaminated’ by the growth of aragonite crystals within it. F) Carbonate tube in transverse section, Ganigobis seep deposits, Namibia, Late Carboniferous (∼302 Ma), showing very similar textures to the tube in E. Scale bars: A = 10mm, B = 1mm, C = 2mm, D = 10mm, E = 100µm, F = 100µm.

Establishing an unambiguous fossil record for Siboglinidae is difficult because the characters that define the family and the contained taxa are based on soft tissues, and these soft tissues are not preserved in the geological record. However, the vestimentiferans, *Sclerolinum* and frenulates produce chemically stable tubes formed of a complex of proteins with inter-woven beta chitin crystallites (e.g., [45], [82]). The tubes of most frenulates and *Sclerolinum* are small (usually only a few mm or less in diameter) and thin-walled (e.g., [83]), and thus have a poor preservational potential in the fossil record. By contrast, many vestimentiferan tubes are large (up to 40 mm in diameter) and robust, often having thick tube walls. Furthermore, vestimentiferans mostly live in environments where rapid mineralization occurs, including carbonates at seeps and sulphides at vents. Thus, vestimentiferan tubes might be expected to have better preservation potential than those of frenulates and moniliferans. Indeed, modern *Ridgeia piscesae* tubes at vents on the Juan de Fuca Ridge can be rapidly overgrown by initial barite and amorphous silica mineralization, which are later replaced by Fe, Zn and Cu sulphides during incorporation into growing sulphide chimneys [84]. A similar pattern of rapid mineralization of vestimentiferan tubes at seeps is found on the Congo deep-sea fan where some posterior ‘root’ tubes of *Escarpia southwardae* are partially to completely replaced by the carbonate mineral aragonite [85], [86]. This replacement occurs from the outside of the tube wall inwards and leaves fine-scale relict textures of the original organic tube wall (Figure 5e). Similar carbonate replaced vestimentiferan tubes are known from seeps in the Gulf of Mexico and Eastern Mediterranean. The oldest fossil attributed to siboglinids is *Hyolithellus micans* from the Middle Cambrian (∼500 Ma), based on tube morphology and the probable presence of chitin in the organic component of the tube wall [87], [88]. However, subsequent authors have not followed this interpretation and attribute phosphatic walled *Hyolithellus* tubes to an unknown extinct order of animals (e.g., [89]). Slightly younger tubular fossils from Palaeozoic (542–251 Ma) hydrothermal vent and cold seep deposits have been formally and informally described as vestimentiferan tubes. Those from the vent deposits (e.g. the Silurian [∼440 Ma] *Yamankasia rifeia* and Devonian [∼393 Ma] *Tevidestus serriformis*) are large (up to 39 mm in diameter) external moulds formed by thin layers of pyrite, often preserving fine details of the external tube wall, including faint longitudinal striations, concentric growth lines and flanges [90]. The tubular fossils from the seep deposits (e.g. the Devonian [∼395 Ma] Hollard Mound and Carboniferous [∼302 Ma] Ganigobis Limestone) are formed of carbonate and have distinctive concentrically laminated tube walls, often showing ‘delamination’ structures (Figure 5f) [85], [91]. These taphonomic (i.e. preservational) features, which are identical to those seen in modern carbonate, replaced vestimentiferan tubes (Figure 5e).

Assigning these Palaeozoic vent and seep tubes specifically to the vestimentiferans raises a phylogenetic problem, because they are considerably older than the divergence estimates of the vestimentiferans from the frenulates based on mitochondrial cytochrome c oxidase subunit 1 (mtCO1), 18S rRNA and 28S rRNA gene studies [35], [70], [79]. These studies suggest that the origin of the vestimentiferans was less than 100 million years ago (i.e., Early Cretaceous), leaving a gap of about 300 million years between this date and the Silurian vent fossils. One explanation is that the Palaeozoic vent and seep tube fossils could represent earlier stem-group siboglinid lineages that are not ancestral to the extant vestimentiferans [81], another explanation is that the fossil tubes are not vestimentiferans (or even siboglinids) and could be fossils of other, possibly extinct, tube forming worms [70], [92]. It may also be the case that gene substitution rates are variable and hence the molecular dates are inaccurate; further work to calibrate the molecular clock in siboglinids is clearly needed.

A few fossil tubes from the Mesozoic (251-65 Ma) and Cenozoic (65-0 Ma) have also been formally described as siboglinid tubes. *Adekumbiella durhami* [93] is a small tube from late Eocene (∼37 Ma) bearing some resemblance to frenulate tubes. The Neogene (23-3 Ma) *Palaeoriftia antillarum* is a large calcareous smooth tube with few features [94]. Tunnicliffe [95] questioned the interpretation of this fossil as a vestimentiferan due to incompleteness of the specimens. Tubular fossils from the early Jurassic (∼185 Ma) Figueroa hydrothermal vent deposit have been assigned to the vestimentiferans [96]. These latter tubes share many morphological similarities with tubes from the younger Upper Cretaceous (91 Ma) Cypriot hydrothermal vent deposits [97], being external moulds of pyrite preserving an ornament of irregularly spaced flanges, concentric growth lines and longitudinal wavy striations with periodic bifurcations and plications where they cross the growth lines (Figure 5a,b) [96]. Identical longitudinal ridges can be seen in the tubes of modern vestimentiferan tubes, particularly at the anterior ends, in both vent (Figure 5c) and seep species (e.g., [96], fig. 8.8–10). Little et al. [96] took this to be a useful character to separate vestimentiferan from frenulate and moniliferan tubes, as neither of the latter groups are known to have this feature. Indeed, many frenulate tubes have distinctive regular constrictions along their length, giving them a ‘bamboo cane’-like morphology (e.g., [83], [96], fig. 8.11). Tubular fossils are also common in Mesozoic and Cenozoic cold seep deposits ([85], table 1, and references therein), some of which are undoubtedly of serpulid origin. However, most (e.g. Figure 5d) are morphologically similar to the modern carbonate replaced vestimentiferan tubes studied by Haas et al. [86] and some of the Palaeozoic seep fossil tubes in having concentrically laminated tube walls, often with ‘delamination’ structures (Figure 5f). Unfortunately this preservation style means that fine scale external ornament is not seen in these fossil cold seep tubes.

Although the majority of the fossil tubes from Mesozoic and Cenozoic seeps and vents are younger than the 100 Ma maximum molecular estimate for the origin of the vestimentiferans, it is difficult to be certain that these fossils are of vestimentiferan origin. The concentrically laminated tube walls with ‘delamination’ structures of the fossil cold seep tubes are a taphonomic feature, not a definitive morphological character, and thus, theoretically, could be a result of the calcification of any multi-layered organic-rich (and probably chitinous) tube (including those of frenulates and *Sclerolinum*) [92]. Nonetheless, this preservational pathway has so far only been proven in the seep vestimentiferans (cf. [92]). The external ornament of longitudinal wavy ridges of the Mesozoic vent fossil tubes (Figure 5a,b) is identical to that seen on all modern vestimentiferan tubes, and not frenulates and *Sclerolinum*, so at present these seem to be among the best candidates for proving a vestimentiferan fossil record, which may thus go back 185 million years. As can be seen above, the fossil record of the frenulates and *Sclerolinum* is considerably poorer and very few fossils may be even tentatively assigned to these siboglinid clades.

Although entirely soft bodied, most species of *Osedax* bore into whale bone [25], [41] and these borings have the potential to be recognized in the fossil record as a proxy for *Osedax* [98]. Indeed, recently borings in Oligocene (∼30 Ma) whale bones from Washington, USA have been interpreted as *Osedax* borings [99]. If correct this would constitute the oldest fossil record of this clade and the age is roughly the same as the first major radiation of whales, which strengthens the idea of an evolutionary link between *Osedax* and its main modern substrate [42].

### How did siboglinids evolve?

#### Adaptation 1: habitat and endosymbiosis

Insights into how siboglinids evolved can initially be derived from examining where these organisms live and commonalities in the physical and chemical parameters of those habitats. The hydrothermal vent habitat of many vestimentiferans is often characterised as an ‘extreme environment’, where organisms must live on the side of mineralized hydrothermal chimneys in which hydrogen sulphide enriched fluids emanate at temperatures of up to 400°C. However, not all vents are like this, in particular many are characterised by more diffuse flow regimes and lower temperatures. In some cases, fluid flow may be through sediments and the organisms that are normally found on hard substrates must cope with this sedimentation. At cold seeps, siboglinids are almost always living within a sedimented environment, although hard substrates do form through carbonate precipitation. Frenulates are also found in sedimented environments, in the anoxic muds beneath organically-enriched regions, although sulphide levels are generally lower than at vents and seeps. Finally, *Osedax* are found living on whale bones which may or may not be sitting on the sediment.

An important commonality in all these habitats is a reduction-oxidation (REDOX) boundary. Living at the REDOX boundary, vent, seep and anoxic mud siboglinids fuel their bacterial symbionts with oxygen, sulphide and carbon dioxide via some unique adaptations to their circulatory system [45]. Bacterial symbionts then fix CO_2_ into organic molecules using sulphide as the energy source [100], [101]. At the strange whale-bone habitat of *Osedax*, less is known about the chemical milieu; the bacterial endosymbiosis and the nutritional pathways are not yet fully understood. Nevertheless, a REDOX boundary and high levels of sulphide are also present at whale bones [102].

**Figure 6:**
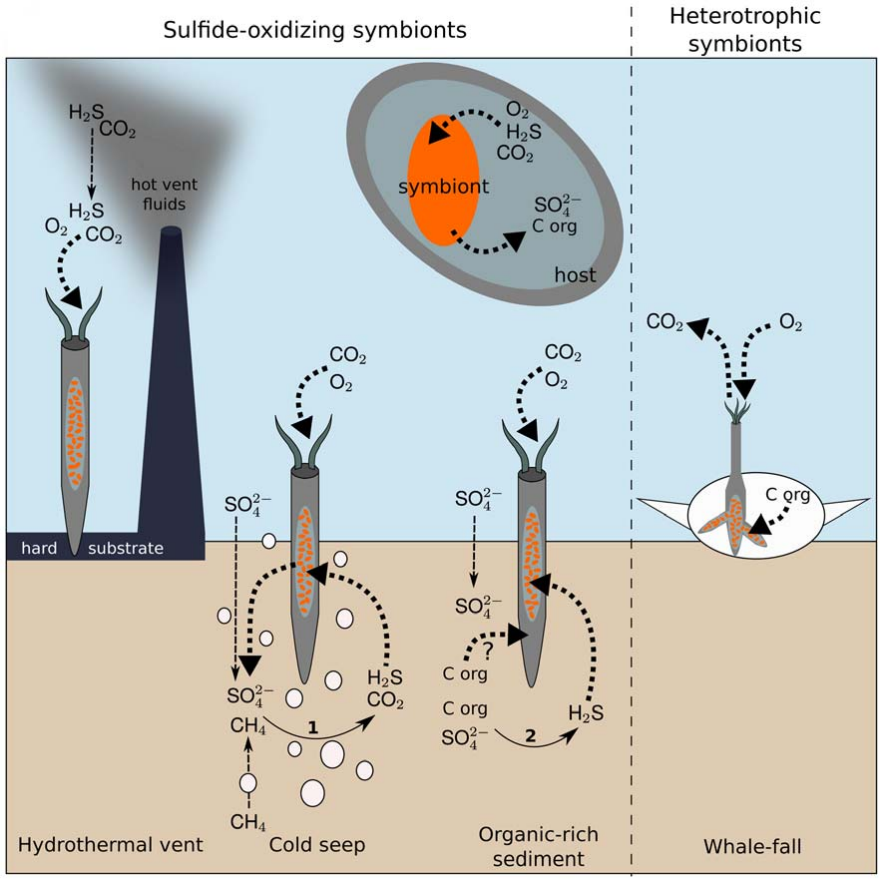
Sources of sulphide and respiratory pathways at contrasting habitats in siboglinid tubeworms. At hydrothermal vents, sulphide is produced through the inorganic reaction of sulphate with geothermal energy. By contrast, sulphide has a microbial origin at cold seeps, organic-rich sediments, and whale-falls. At cold seeps, the source of sulphide is the anaerobic oxidation of methane coupled to sulphate reduction. At organic-rich sediments, sulphide is produced during the anaerobic degradation of a range of organic compounds. At whale-falls, although sulphide is produced, *Osedax* worms are thought to rely only on heterotrophic digestion of bone by the endosymbionts. The trophosome (light grey) houses endosymbiotic bacteria (orange ovals). White open circles represent methane and hydrocarbon seepage. Full arrow = reaction, dashed arrow = diffusion, and dotted arrow = acquisition or excretion by the host/symbiont.

Siboglinids living in different environments have evolved adaptations to exploit differences in food and sulphide (or in some cases methane) availability. Whereas vestimentiferans living on hydrothermal vent chimneys absorb sulphide through a branchial plume that extends up to 2 m into the water column [103], vestimentiferans living in cold seeps obtain sulphide from the sediment, across the wall of the buried tube [104] (Figure 6). Frenulates, notwithstanding some exceptions, are found mainly in organic-rich, reduced sediments. Because frenulates can transport dissolved organic matter across their tube and body wall [105], sulphide is presumably transported across the thin tube that is buried in the sediment, but data supporting this are scarce. In the case of the frenulate *Siboglinum poseidoni*, methanogenesis is reported [106]. Sulphide levels or uptake location have not yet been investigated for *Sclerolinum* species, and for *Osedax*, the current evidence suggests that the endosymbionts are consuming collagen or lipids directly from bones rich in these energy sources [54].

A crucial adaptation in the evolution of siboglinids appears to be a unique circulatory system that allows these chemicals to be delivered to the symbionts. Sulphide and oxygen are transported from the site of uptake (e.g. the branchial plumes or body walls) via haemoglobin molecules that are freely dissolved in their blood or in the coelomic fluid surrounding the blood vessels [107]–[109]. These haemoglobin molecules exhibit some unique properties. Three and two types of haemoglobin have been identified in vestimentiferans [109] and *Sclerolinum* [110], respectively. One is a hexagonal bilayer haemoglobin (HBL-Hb) that is capable of binding oxygen and sulphide simultaneously and reversibly [100], [109], enabling the animals to transport and store both substances in large quantities while minimizing autoxidation and toxic effects [19]. A second type of haemoglobin detected in Siboglinidae is a ring-Hb that has been found in Vestimentifera, *Sclerolinum*, and Frenulata. Although sulphide binding has not been demonstrated for the ring-Hb, it has an extremely high affinity for oxygen [107], [110], [111] that enables the worm to take up and transport large amounts of oxygen while maintaining low internal dissolved O_2_.

Equally important to adaptations within the circulatory system are the bacterial endosymbionts that are thought to provide the majority of energy to the hosts. Considering the diversity of both siboglinid worms and the habitats that they occupy, the existence of considerable bacterial endosymbiont diversity is perhaps unsurprising. Siboglinids engage in an obligate and persistent association with a numerically dominant phylotype of Gammaproteobacteria, referred to here as the “primary endosymbiont” ([53], [58], [59], [112], [113], but see [54], [114], [115]). Major siboglinid groups (i.e., frenulates, vestimentiferans/*Sclerolinum*, and *Osedax*) each associate with a different bacterial clade, reflecting host-symbiont specificity at higher taxonomic levels [57]–[59], [116], [117]. In vestimentiferans and *Sclerolinum* specifically, primary endosymbionts are two closely-related clades of chemoautotrophic bacteria within the Leucothrix-Methylococcaceae cluster. Information on symbiont diversity is more limited for frenulates. The three frenulate species examined to date harbour primary endosymbionts within a monophyletic clade of thiotrophic Leucothrix-Methylococcaceae Gammaproteobacteria [56]–[59]. Despite their apparent metabolic similarity to the vestimentiferan/*Sclerolinum* symbionts, the frenulate symbionts are phylogenetically distinct from symbionts of other siboglinids [57]–[59]. Notably, one species of frenulate, *Siboglinum poseidoni*, harbours a methanotrophic endosymbiont [106], [118] of unknown phylogenetic affinity. Finally, primary endosymbionts of *Osedax* belong to the Oceanospirillales cluster [51], [54], [55], a diverse bacterial group known for heterotrophic aerobic degradation of complex organic compounds. The role of the endosymbionts within *Osedax* is not clear, but they are hypothesized to provide nutrition to their hosts via the degradation of bone collagen [54].

In addition to the primary endosymbiont, bacterial consortia (referred to here as the “microflora”) have been found in some siboglinids. These additional bacterial types consist of multiple bacterial lineages, including Alpha, Gamma, and Epsilonproteobacteria as well as members of the Bacteroidetes (e.g., [51], [54], [55], [113]–[115]). The microflora typically occur at lower relative abundance compared to the primary endosymbiont and may not even be located within the host trophosome [54], [55], [57], [113]. The nutritional contributions of these bacteria to their siboglinid hosts remain unknown and offer fertile ground for future research.

In terms of symbiont acquisition, despite the obligate nature of this mutualism, horizontal uptake of bacteria from the surrounding environment or co-occurring hosts is used [119], [120]; but see [121]. Available evidence supporting horizontal transmission as the primary mode for establishment of siboglinid symbioses includes: (1) a lack of symbionts in worms' gonadal tissues or larvae [13], [55], [122]–[124], (2) the presence of the motility-related flagellin gene in the vestimentiferan endosymbiont genome [117], [125], (3) the detection of highly similar bacterial phylotypes (based on 16S rRNA sequences analysis) in host and in the external environment [112], [126]–[129], (4) the presence of heterotrophic metabolic pathways in the vestimentiferan endosymbiont that are not expressed *in hospite* [117], (5) direct confirmation of horizontal transmission in *Rifta pachyptila* [26], and (6) the absence of reciprocal phylogenies (i.e., co-evolution) between host and symbiont [112], [130], [131]. Thus, following a non-symbiotic larval stage, siboglinids must establish a new symbiosis each generation in order to survive. Despite the risk of failing to acquire an appropriate symbiont, horizontal transmission presumably enables the host to acquire a bacterial phylotype adapted to the local environmental conditions (e.g. sulphide concentration [60] or bone degradation stage [132]).

Following acquisition from the environment, bacterial symbionts migrate to the trophosome in some vestimentiferans [26], [47]. Although it has previously been hypothesized that symbionts were acquired from the environment during the trochophore larval stage [32], [133], recent work indicates that vestimentiferans are colonized by bacteria after larval settlement and development of a juvenile worm [26]. Remarkably, Nussbaumer et al. [26] showed that symbionts enter the host through the epidermis during a symbiont-specific selective infection process and subsequently migrate into a mesoderm tissue that will develop into the trophosome. Once the trophosome is well established in juveniles, the infection ceases at the same time as apoptosis of skin and other non-trophosome tissues. The timing (larval or post settlement) and mechanism of symbiont acquisition from the environment are not known for other siboglinid groups. In *Osedax*, it has been proposed that infection would not be limited in time but continuous throughout the worm life, with symbionts infecting new root tissue as it grows into whale bones [55].

The obligate symbiosis in siboglinid tubeworms at deep-sea vents, seeps and whale-falls is a most remarkable biological adaptation. Still, many questions remain unanswered. In particular, the winnowing processes that occur from infection by the symbionts to colonization by the primary endosymbiont are unknown. Unfortunately, symbiosis has only been investigated in a handful of siboglinid species. The question of nutrition in siboglinids has consumed research in this area, but results have been difficult to come by. For the first few decades, a handful of clever experimental studies suggested the paradigm of DOM uptake across the body wall. The following few decades have assumed that endosymbioses plays the primary role. Either way, the presence of luxuriant fields of giant tubeworms on the sulphide chimneys of the East Pacific Rise, without mouth or gut and reliant only on the chemistry of the moment to survive remains one of the more interesting possibilities of evolution.

#### Adaptation 2: reproduction and dispersal

The majority of deep-sea polychaetes live in the vast tracts of sedimented mud that dominate the abyssal seafloor. Habitat availability and stability are not, in general, a problem for organisms that can live on approximately 60% of the planet's surface. In contrast, many siboglinid habitats, including hydrothermal vents, cold seeps and whale-falls are extremely small and isolated habitats, often separated by 100s to 1000s of km. The evolutionary innovation of symbiosis that allowed siboglinids to invade and radiate on sulphide-rich ‘island’ habitats in the deep-sea must also have been coupled with equally innovative life-history strategies to ensure that the reproductive propagule can locate and colonize the “needle” in the oceanic “haystack”.

While difficult logistics have so far precluded intensive time-series studies of the reproductive activity of any siboglinid species, much has been learned about the reproductive ecology through “snap-shot” analyses of, for example, gametogenic condition, population structure and population genetics [134]–[136]. Similarly, studies of early development based on spawning wild-caught individuals have provided insights into dispersal of all siboglinid clades [23], [24], [124], [135], [137]. Despite these increases in available data, very little is known about reproduction and dispersal of siboglinids in an evolutionary context.

Life-history theory predicts traits that maximize fitness of an organism in the particular environment where it lives. Therefore, differences between siboglinid habitats are expected to have a role in the evolution of life-history traits, including fecundity, breeding strategy and developmental mode. At present, we do not have estimates of lifetime fecundity for any siboglinid. However, instant fecundity data suggest that the Vestimentifera and *Osedax* have generally higher fecundity than Frenulata ([124]; Hilário pers. observ.). Although this could be related to body size (since small animals are expected to produce a small number of large eggs [138]), it is most likely related to the energy available in the environment and the insular and/or ephemeral nature of hydrothermal vents, cold seeps and whale falls. Siboglinids living in vents, seeps and whale falls have access to sufficient energy to invest in high fecundity, which in turn allows them to exploit these isolated and sometimes ephemeral habitats.

**Figure 7:**
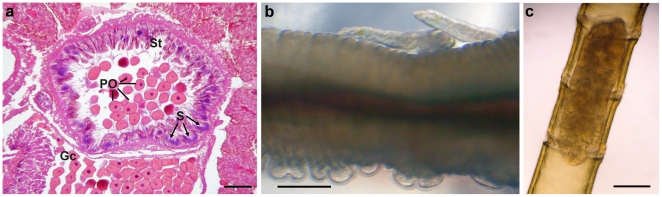
Life-history traits of Vestimentifera, *Osedax* and Frenulata. A) Histological section through the spermatheca of *Riftia pachyptila* (Vestimentifera) (Gc = Gonocoel, PO = Primary oocyte, S = Clusters of spermatozoa, St = Spermatheca) (from [135]). B) Two live males on the trunk of a female of an undescribed species of *Osedax* recovered in Antarctic waters. C) Brooding larva inside the tube of *Siboglinum* sp. (Frenulata). Scale bars: A = 200 µm, B = 100 µm, C = 500 µm.

Fertilization is assumed to be internal for all siboglinid clades (no information is available for *Sclerolinum*). To further facilitate fertilization, Vestimentifera females store sperm in a spermatheca until eggs are mature (Figure 7a, [135]). *Osedax* have evolved a specialized strategy to ensure reproductive success; females host dwarf males in their tubes assuring sperm availability (Figure 7b, [25], [124]). Therefore, vestimentiferans and *Osedax* both utilize strategies in environments where periodic cues for gametogenesis and spawning synchrony are limited [139] and mate acquisition is not guaranteed.

Following fertilization and embryogenesis, planktonic larvae develop. Larval dispersal duration and distances are intuitively most likely related to habitat isolation. In vestimentiferans, small, yolky and slightly buoyant eggs develop into non-feeding trochophore larvae that are thought to disperse in the plankton for up to several weeks [23], [24]. For instance, larvae of the vent species *Riftia pachyptila* are estimated to disperse more than 100 km over a 5-week period [24]. Whilst the vent and seep habitats of vestimentiferans are restricted geographically to areas such as mid-oceanic ridges and continental margins, the whale-fall habitats of *Osedax* may occur anywhere throughout the world's oceans where whales are present. As a result, *Osedax* are hypothesized to have shorter dispersal times and distances than vestimentiferans [124]. Although no estimates exist for larval dispersal distances and duration of Frenulata, it is known that some species incubate eggs in their tubes until settlement stage (Figure 7c) whereas others have planktonic larvae, although the latter have never been reared [48]. Brooding is presumably favoured by natural selection on continuous habitats, such as anoxic sediments that are almost continuous along continental margins, as the great expanses of suitable substratum make colonization of new habitats unnecessary. Insufficient sampling of frenulates, however, does not allow robust comparisons between habitat isolation and developmental mode.

A detailed phylogenetic analysis of Siboglinidae is needed to provide a framework for understanding the evolution of life-history traits in the group. However, it does appear that the various reproductive strategies found in siboglinids are related to environmental conditions. Notwithstanding possible exceptions, the overall rank order of fecundity and dispersal distance of siboglinids is: Vestimentifera>*Osedax*>Frenulata corresponding to the degree of transience and isolation of the habitats occupied by these groups. The placement of *Sclerolinum* in this rank remains unknown, as no reproductive data are currently available.

## Discussion

The two questions posed by this review are when and how these worms evolved. How were these metazoans able to make the transition to an extreme habitat, apparently high in toxic sulphide and competing mats of free-living bacteria? When did this happen in Earth's history? Was it driven by the geological formation of spreading centres and hydrocarbon seeps? Or was there a long gap between the availability of the habitat and the biological adaptations necessary to colonise it?

These questions are not easy to answer, particularly so when it has taken over eighty years of detailed research even to determine the taxonomic placement of siboglinids. When confronted with a biological ‘oddity’, such as giant red tubeworms on a deep-sea volcanic vent, taxonomy is the first tool to be brought out. At several moments in the scientific history of siboglinid research, it has been a key taxonomic paper – often published in a high-impact journal – that has spurred research in the field. It is rare that deep-sea worm genera such as *Riftia* or *Osedax* are described in the pages of *Nature* or *Science*. However, in these cases, research into these animals was stalled until the names were published. It was the formal taxonomic publication, the creation of a compelling name and common language that allowed researchers to finally start linking together work on the biology of these unusual animals.

Attached to the name is often a hypothesis of an organism's closest relatives. For siboglinids, this has challenged taxonomists, anatomists and evolutionary biologists. Only molecular genetics have provided recent convincing, consistent character sets, although with hindsight, the morphological clues were always there. Molecular and morphological phylogeny studies now place frenulates in a basal position with vestimentiferans and *Sclerolinum* nested within this larger clade. Among vestimentiferans, vent species are nested within the clade of seep-dwelling species, which has led several authors to suggest that siboglinid evolution originated in soft substrates and progressed through to the species that live on sulphide-rich hydrothermal vents [35], [44], [60], [140]. This seemingly ordered trend has been complicated by the discovery of the *Osedax* clade, specialist on whale bones and using heterotrophic rather than chemoautotrophic symbionts.

The evidence so far suggests that the last common siboglinid ancestor was likely either symbiotic or pre-adapted to symbioses with gamma proteobacteria. Given that there are, so far, only four known lineages of siboglinids and that symbionts within a major host lineage seem to be related, there are a limited number of alternative scenarios for the evolutionary origins of this symbiosis. The scenarios include: (1) an aposymbiotic ancestor, with endosymbiosis being established more than once independently in major siboglinid lineages, (2) a symbiotic ancestor that gave raise to major lineages that experienced switches in primary endosymbiotic phylotype, or (3) an ancestor that housed a consortia of bacteria and as major lineages emerged so did specialization in primary phylotype among lineages.

**Figure 8:**
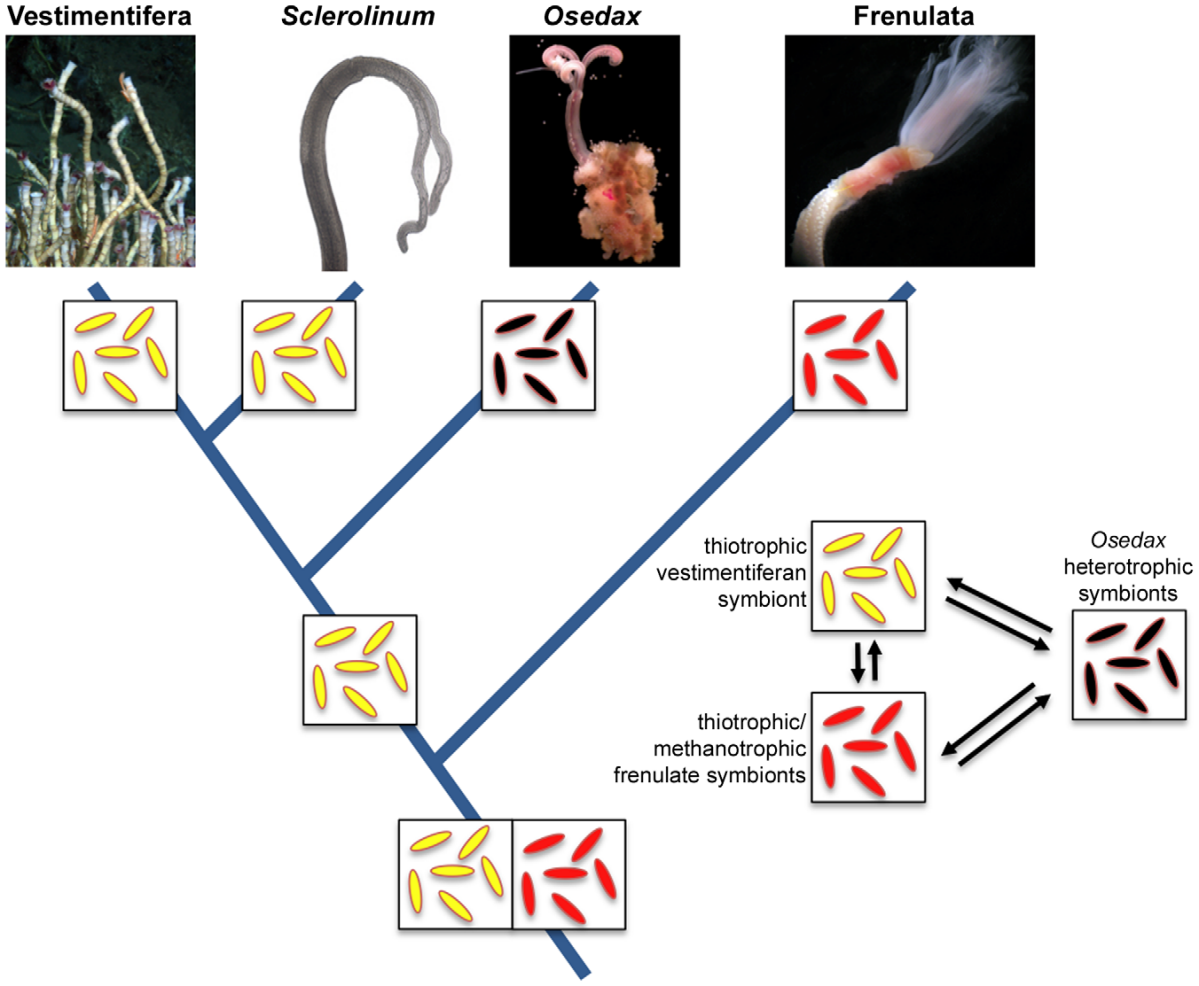
An evolutionary scenario for the origin of the major siboglinid clades and their respective symbiont. Note that the sister-group relationship between *Osedax* and the vestimentiferan-*Sclerolinum* clade is currently only weakly supported. In this scenario, the putative siboglinid ancestor possessed chemoautotrophic symbionts that have been secondarily lost in *Osedax* and replaced by a heterotrophic symbiont. Images courtesy of DT, KH, Kevin Fielman and Scott Santos (vestimentiferan), Irmgard Eichinger (*Sclerolinum*).

Available data support limited concordance between host and symbiont phylogenies. For example, although monophyletic clades of symbionts for vestimentiferans, *Sclerolinum*, frenulates, and *Osedax* are resolved, the deeper relationships between clades are not well resolved (Figure 3). Furthermore, the sister group relationship between *Osedax* and vestimentiferan hosts is tentatively supported in the phylogenetic analysis by Rouse et al. [25] but less in Glover et al. [41]. However, if one assumes that it is a greater number of evolutionary steps to transition from a chemoautotroph symbiont to a heterotroph symbiont than it is between two different types of chemoautotroph symbiont, parsimony arguments support a siboglinid ancestor with two possible chemoautotroph symbionts and the secondary loss of chemoautotrophy in *Osedax* (Figure 8).

If, as speculated, the evolution of host lineages may be driven by an evolutionary trend in the REDOX potential of the environments that host worms inhabit, this hypothesis would also explain why, from an evolutionary physiology point of view, the host would switch or specialize its symbiont community. As the host moved into new environments, different lineages of Gammaproteobacteria would allow more successful exploitation of the REDOX conditions within that environment. For example, consider that sulphide is available at whale-falls [102], whalebones often become sedimented, and that some species of *Osedax* have been found to specialize on bones buried in sediment [132]. An ancestor of *Osedax* may have contained a typical thiotrophic endosymbiont form that utilized sulphide rich sediment around whalebones. However, the energy reserves in the collagen of whalebones were a large untapped energy source offering a great selective advantage to, and rapid evolution of organisms that could utilize it. Thus, the hypothetical thiotrophic *Osedax*-ancestor made the evolutionary transition to heterotrophy. One piece of evidence in support of this hypothesis is that vestimentiferans, with thiotrophic symbionts, have been recorded occasionally in sediments containing whalebones, although never ecologically dominant [141]. It may have been that this type of occasional habitat colonization, with overlapping sulphide conditions, was the necessary evolutionary step in the origin of *Osedax*.

Independently of how siboglinids evolved, their evolutionary age is one of the most intriguing subjects of chemosynthetic ecosystems biology. For now we are unable to confidently delineate a timeframe during which Siboglinidae split from its polychaete relatives or the age of the most recent common ancestor between clades. The fossil record suggests a Mesozoic or even Palaeozoic origin, which largely disagree with molecular divergence phylogenies, that indicate a much younger origin [70], [92], [96]. This discrepancy raises several questions about the interpretation of both the molecular and fossil data. However, to investigate the origins and ages of siboglinids in relation to their habitat the fossil record may provide valuable clues and validate hypotheses of divergence times such that *Osedax* origin coincided with that of its main modern substrate – the large oceanic cetaceans (e.g. [42]).

### Conclusion and Future Directions

The circular story of Siboglinidae systematics is, as Pleijel et al. [63] have put, “one of humbleness… a reminder that we are all likely to make mistakes”. None of the four major lineages of siboglinids have proved easy to sample, identify, classify or study. For almost 80 years, from their discovery in 1914 to the first molecular phylogenies in the 1990s, there was disagreement over what the frenulate pogonophore worms actually were. The more recently discovered vestimentiferan tubeworms also proved difficult to understand, despite their greater size. Even the most recently discovered group, *Osedax*, took over 10 years to be identified and described, from the first observations of small gelatinous tube worms attached to whale bones recovered from the Oregon subduction zone in 1994 (Dr. Eve Southward, pers. comm.) to the description and classification of the genus in 2004 [25].

Given the known diversity of siboglinds, one obvious issue in the study of siboglinid history is the lack of sampling among frenulate taxa. The fossil record is very poor and only 5 out of 140 described frenulate species have been examined in molecular phylogeny studies. Sampling constraints associated with the small size on the individuals, a shortage of taxonomic expertise, and the fact that for a long time specimens were routinely fixed in formaldehyde, which is incompatible with most molecular biology techniques, have all contributed to the current situation of frenulates being the least-studied group of siboglinids. The lack of sampling among frenulate taxa has, in the last few years, stimulated new collections and research. Additional morphological and genetic information on frenulates is in the process of being disclosed [57], [142], [143].

In spite of the spectacular discoveries and extraordinary advances made in recent years the placement of siboglinids among the annelid tree is still poorly resolved and many other questions concerning the evolution and ecology of siboglinids remain unanswered. New challenges are presented to scientists at a daily basis. Yet many siboglinids live in relatively inaccessible environments and therefore understanding the larger picture of siboglinid evolution in relation to their habitat requires a concerted effort into deep-sea exploration. Only a small fraction of the global ridge system (∼65 000 km) and of the vast continental margin regions have been explored. We believe that the exploration of new chemosynthetic environments, on planet earth and perhaps beyond, will include the discovery of new species capable of ecological and physiological attributes that cannot yet be imagined.
